# Associations of physical activity levels with fatigue in people with inflammatory rheumatic diseases in the LIFT trial

**DOI:** 10.1093/rap/rkae106

**Published:** 2024-08-24

**Authors:** Stuart R Gray, Alexander H K Montoye, Joseph D Vondrasek, Sylvia Neph, Stefan Siebert, Lorna Paul, Eva M Bachmair, Neil Basu

**Affiliations:** School of Cardiovascular and Metabolic Health, University of Glasgow, Glasgow, UK; Integrative Physiology and Health Science Department, Alma College, Alma, MI, USA; Integrative Physiology and Health Science Department, Alma College, Alma, MI, USA; Integrative Physiology and Health Science Department, Alma College, Alma, MI, USA; School of Infection and Immunity, University of Glasgow, Glasgow, UK; Department of Physiotherapy and Paramedicine, Glasgow Caledonian University, Glasgow, UK; Aberdeen Centre for Arthritis and Musculoskeletal Health, University of Aberdeen, Aberdeen, UK; School of Infection and Immunity, University of Glasgow, Glasgow, UK

**Keywords:** physical activity, fatigue, inflammatory rheumatic disease

## Abstract

**Objectives:**

The overall aim of the current study was to quantify physical activity levels in inflammatory rheumatic diseases (IRDs) and to explore their role in fatigue.

**Methods:**

We conducted a secondary analysis of data from the Lessening the Impact of Fatigue in IRDs (LIFT) trial of the personalized exercise program (PEP) intervention for fatigue. Participants with IRDs were recruited from 2017 to 2019 and the current analysis used fatigue, measured by the Chalder Fatigue Scale (CFS) and the Fatigue Severity Scale (FSS), and accelerometer measured physical activity data collected at baseline and at the 6-month follow-up. Physical activity levels were quantified and associations with fatigue and effects of PEP investigated.

**Results:**

Of the 337 included participants, 195 (68.4%) did not meet the current recommendations for moderate–vigorous physical activity (MVPA). In baseline cross-sectional analysis, many dimensions of physical activity were associated with fatigue. After mutual adjustment, overall physical activity (vector magnitude) was associated with CFS [−0.88 (95% CI −0.12, −1.64)] and distribution of time spent at different activity intensities was associated with FSS [−1.16 (95% CI −2.01, −0.31)]. Relative to usual care, PEP resulted in an increase in upright time, with trends for increases in step count and overall physical activity. People who increased overall physical activity (vector magnitude) more had greater improvements in CFS and FSS, while those who increased step count and MVPA more had greater improvements in FSS.

**Conclusion:**

Increasing physical activity is important for fatigue management in people with IRDs and further work is needed to optimize PEPs to target the symptoms and impact of fatigue.

**Trial registration:**

ClinicalTrials.gov (http://clinicaltrials.gov), NCT03248518.

Key messages

- Fatigue remains a common and highly deleterious symptom in people with inflammatory rheumatic diseases.
- This study has shown that objectively measured physical activity levels are associated with fatigue.
- Our personalized exercise program resulted in modest improvements, with increases in physical activity related to improvements in fatigue.

## Introduction

Inflammatory rheumatic diseases (IRDs) (e.g. RA, axial SpA and SLE) are common and make a major contribution to the global disability burden [1]. Despite recent changes in IRD treatment, the symptom of fatigue remains highly prevalent. Approximately 80% of people with IRDs report significant fatigue [2] and >70% consider the symptom to be as great a burden as pain [3]. Additionally, fatigue is a major contributor to low quality of life and work disability [4].

There are no effective pharmacological treatments for fatigue related to IRDs [5]. Cross-sectional data indicate that higher levels of physical activity are associated with lower fatigue levels in the general population and in people with RA [6–11], but these data cannot demonstrate causation or directionality. We recently conducted LIFT, which included an investigation of whether a personal exercise program (PEP) was effective in reducing fatigue in people with IRDs [12]. This program was designed to gradually increase the level and intensity of participants’ exercise and/or physical activity to at least the levels recommended by national guidelines (150 min of moderate-intensity physical activity per week) [13]. After 6 months of PEP, the severity and impact of fatigue was reduced, and these changes were sustained after a 6-month follow-up [14]. This finding highlighted the importance of exercise and physical activity in the management of fatigue in IRDs.

In LIFT, PEP was tailored to each participant’s needs, with the general goal of meeting physical activity recommendations while avoiding boom-and-bust patterns of activity. However, we did not investigate the factors (i.e. intensity, time, distribution) of physical activity that were most effective for improving fatigue in this population. Importantly, ‘physical activity’ is a broad term that encompasses numerous modalities and intensities. Recent advancements in physical activity measurement have moved beyond simple and older measures of step count or light/moderate/vigorous intensity activity, so new metrics such as the distribution of physical activity intensity across the activity profile can be assessed. One such metric, the intensity gradient, is a measure of how much time people spend at different intensities of physical activity [15, 16]. A negative intensity gradient reflects more time performing lower intensity exercise compared with high intensity, with a less negative gradient reflecting an even distribution between the intensities. In the general population, people spend more time performing lower intensity exercise and therefore have a more negative intensity gradient [15]. In previous research on both adolescent girls and in people with type 2 diabetes, the intensity gradient was associated with cardiometabolic risk factors independent of overall physical activity levels [15]. Given this independent association, intensity gradient may also be an important health metric beyond total physical activity. The intensity gradient may provide complementary information to measures of overall physical activity levels. Additionally, the intensity gradient allows us to more fully describe the activity profile of various populations and how activity may be related to health outcomes [15].

With this in mind, we nested an accelerometery substudy into the LIFT, where we measured physical activity using activPAL thigh-worn accelerometers (PAL Technologies, Glasgow, UK). Our original plan (although this was not pre-specified in the study protocol) was to focus on more standard metrics, such as step counts, but with developments in physical activity measurement we were able to add novel metrics such as the intensity gradient. This substudy provides an opportunity to further explore objectively measured habitual physical activity levels and patterns. These devices have previously been validated in people with RA for the measurement of physical activity and sedentary behaviour [17]. Current data, which are primarily based on self-reports, indicate that sedentary time is high and physical activity low in people with IRDs [18–20], which is supported by a small study demonstrating that activPAL-measured sedentary time was higher and physical activity lower in people with RA compared with sex-, age- and BMI-matched control participants [21]. How established objective measures of overall physical activity (e.g. step count), along with complementary dimensional metrics (e.g. intensity gradient), are associated with fatigue in people with IRDs remains to be established.

Therefore, the aims of the current study were to quantify physical activity levels and patterns in IRDs with stratification based on age, disease and sex; investigate associations between IRD fatigue and comprehensive physical activity metrics, including the novel intensity gradient; determine the effects of the PEP on physical activity metrics and explore how changes in physical activity associate with changes in fatigue following the PEP in people with IRDs.

## Methods

The LIFT methods are briefly described here, with full details published separately [12, 14].

### Study design and participants

The LIFT was a multicentre randomized controlled open-label parallel-group trial that recruited participants with stable IRDs who reported fatigue to be persistent (>3 months) and clinically significant [≥6/10 based on the question: ‘Please circle the number that shows your average level of fatigue during the past 7 days on a numerical rating scale of 0 (no fatigue) to 10 (totally exhausted)’]. Participants were randomized to either PEP, cognitive behavioural approaches (CBAs) or usual care (1:1:1 ratio). The current analysis focuses on the effects of PEP, rather than CBAs, compared with usual care, although in baseline analysis all participants were included. Ethical approval was granted by Wales REC 7 (17/WA/0065) and informed consent provided by all participants.

### Procedures

PEP was delivered by physiotherapists working within the National Health Service. Participants were invited to seven one-to-one sessions (up to 45 min per session) over a 14-week period, with a follow-up booster session at 22 weeks. Apart from the first session, which was delivered in person, all other sessions were delivered remotely by telephone. The therapist and participant agreed on goals and developed a PEP with the aim of meeting the physical activity guidelines by increasing the duration and intensity of physical activity while avoiding boom-and-bust patterns [13].

Outcomes were assessed at baseline and 10, 28 and 56 weeks. Outcomes included in the current study were the co-primary outcomes from the LIFT. These were the Chalder Fatigue Scale [CFS; 0 (low)–33 (high)] [22], which assesses the physical and mental symptoms of fatigue, and the Fatigue Severity Scale [FSS; 1 (low)–9 (high)] [23], which measures the impact of fatigue. These distinct measures of fatigue, covering both symptoms and impact, may be influenced to varying degrees by physical activity. Physical activity was measured continuously for a 7-day period at each time point with an activPAL accelerometer worn in a mid-anterior position on either thigh, attached via a waterproof dressing. Age, BMI and sex were recorded. Disease activity was self-reported using a numeric rating scale [0 (not active)–10 (extremely active)] and the presence of comorbidities was recorded via the Charlson Comorbidity Index (CCI) [24].

### Accelerometer data processing

Data were downloaded using the PALbatch software (PAL Technologies, Glasgow, UK), using the default setting of the 24-h protocol, which considers days valid if there is <4 h of non-wear. Standard metrics of step count, activity score, sedentary time, upright time, stepping time, cycling time, lying time, seated transport time, breaks in sedentary time (time transitioning from sedentary behaviours to non-sedentary behaviours) and vector magnitude [$\text{vector}\;\text{magnitude}\;=\;\sqrt{\left(x^{2}+y^{2}+z^{2}\right)}$, where *x*, *y* and *z* are the accelerometer axes] were obtained using the enhanced PAL analysis algorithm (CREA) available within the software. Additionally, the raw accelerometery data were exported to calculate the following physical activity metrics. Moderate–vigorous physical activity (MVPA), the target of most government physical activity guidelines, was calculated as the time spent at an intensity of >80 steps/min, and participants were defined as having insufficient physical activity if they performed <150 min/week of MVPA [25]. More detailed analyses of the data were also conducted in order to understand the movement patterns of participants. Specifically, the vector magnitude data were used to calculate the intensity gradient, a measure of the intensity distribution of physical activity, as described previously [15]. Additionally, the MX metrics, which describe the acceleration (vector magnitude) above which the most active X min are accumulated, as described previously [16] were calculated. The M5 (acceleration in the most active 5 min) and M60 (acceleration in the most active 60 min) were calculated in the current study. MX metrics provide insights into a wearer’s most active portion of the day and provide an alternative way to assess adherence to physical activity recommendations (e.g. if the M60 is above a given MVPA threshold, it is clear that the individual engaged in at least 60 min of MVPA that day), but also to allow researchers to understand the distribution of activity intensity in a more continuous way than the more traditional, cut-point thresholds used to categorize activity intensities as sedentary, light or MVPA.

### Statistical analysis

Variables were compared between participants with RA *vs* those with other IRDs (non-RA), between males and females and between younger (<60 years) and older (≥60 years) participants by independent *t*-tests. Data were split into these categories as we considered these the main variables that might influence participants’ physical activity data. Associations of physical activity metrics (exposures) with FSS and CFS (outcomes) were tested using multivariable linear regression in all participants (PEP, CBA and usual care) at baseline. Due to the high number of potential exposures, we selected the following variables to represent physical activity volume, patterns and sedentary behaviours (step count, vector magnitude, intensity gradient, M5, M60, lying time, sedentary time, upright time and MVPA). The following models were employed: model 1, unadjusted; model 2, adjusted for age, sex, body mass, disease activity score and CCI; and model 3, adjusted for model 2 + vector magnitude when intensity gradient was the exposure and model 2 + intensity gradient when vector magnitude was the exposure. Vector magnitude was chosen as a marker of overall physical activity and the intensity gradient as a marker of activity intensity distribution. Changes in physical activity metrics in the PEP at 6 months were compared with usual care via analysis of covariance (ANCOVA), with baseline values of the physical activity metric included as a covariate. The CBA group was not included in this analysis, as our focus was on the PEP group. The 6-month time point was chosen as the end of the active intervention, as at 12 months the number of participants with valid accelerometer data had decreased to 30 in PEP and 42 in usual care, compared with 45 in PEP and 48 in usual care at 6 months. In the exploratory analysis, the change in CFS and FSS was explored within the PEP group by stratifying the sample by the change in physical activity metrics at the median (low and high change). We then compared CFS and FSS at 6 months between the low and high changes groups, with baseline CFS and FSS scores as a covariate.

## Results

### Study population and basic demographics

A total of 337 participants (225 female and 82 male) were included in the current study, with 191 having RA and 146 non-RA IRDs (including CTD, axial SpA, systemic vasculitis, juvenile inflammatory arthritis and undifferentiated inflammatory arthritis), with the latter grouped together due to low numbers for each condition. Basic baseline demographics are presented in Table 1, with demographics by condition, sex and age (<60 years and ≥60 years) presented in Supplementary Tables S1–S3, respectively (available at *Rheumatology Advances in Practice* online).

**Table 1.** Basic demographic and physical activity variables in participants from the LIFT (*N* = 337)

Variables	Values, mean (s.d.)
Age (years)	57.9 (12.6)
Body mass (kg)	78.5 (16.4)
Male, *n* (%)	82 (24.3)
Steps/day	6959 (3259)
Activity score (MET/s)	83.3 (3.5)
Sedentary time (min/day)	549.4 (110.7)
Upright time (min/day)	325.8 (112.6)
Stepping time (min/day)	91.3 (38.3)
Cycling time (min/day)	0.83 (2.86)
Lying time (min/day)	565.8 (82.2)
Seated transport (min/day)	51.3 (39.9)

### Physical activity metrics

**Figure 1.**
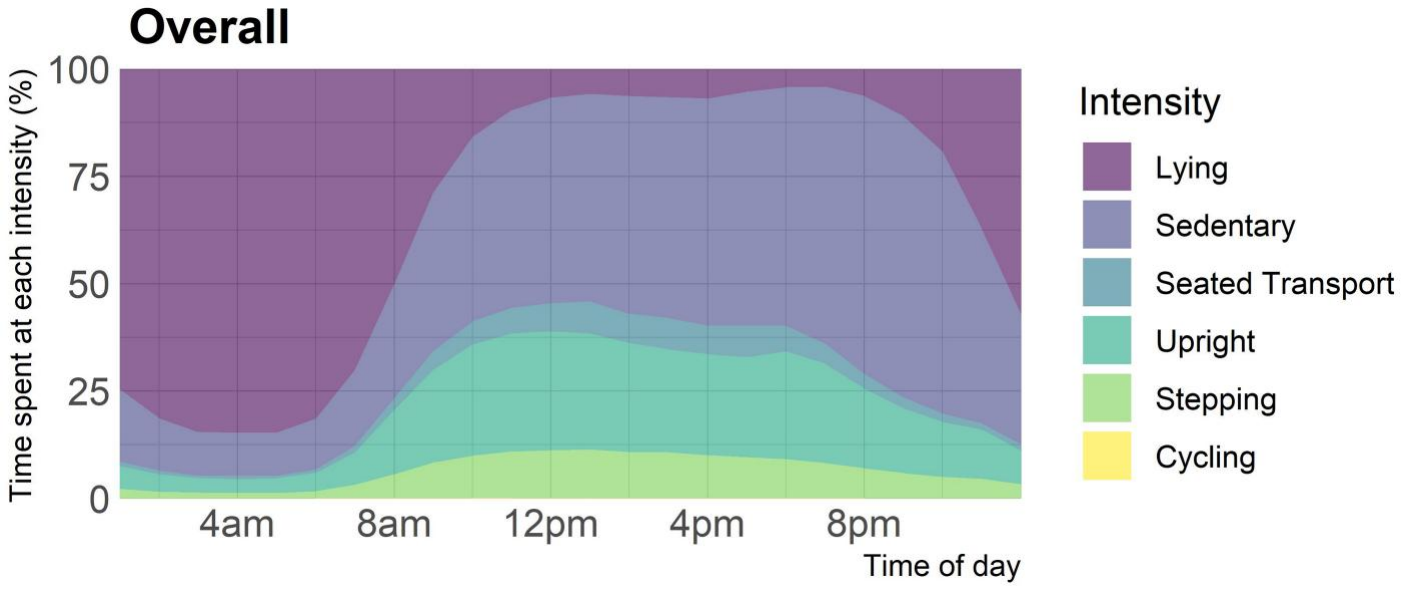
Variation in accelerometer-measured time by activity type in all participants in the LIFT

Baseline overall physical activity metrics are presented in Table 1, with novel physical activity metrics presented in Table 2. Summarizing briefly, in the whole sample, participants took an average of 6959 steps/day and participated in 17.8 min/day of MVPA, with 68.4% of participants being classed as having insufficient physical activity. The same data presented stratified by RA *vs* non-RA, males *vs* females and <60 years *vs* ≥60 years are presented in Supplementary Tables S1–S6 (available at *Rheumatology Advances in Practice* online). To visualize the time spent in each activity across the day we present stacked plots for the overall cohort in Fig. 1 and stratified by RA status, sex and age in Supplementary Figs S1–S3 (available at *Rheumatology Advances in Practice* online). No major differences were seen between the RA and non-RA groups, but some sex and age differences were noted. Women spent less time cycling, although cycling time was low regardless of sex, less time in seated transport, lower overall physical activity (vector magnitude) and lower M60 values. Younger people were more sedentary, spent more time in seated transport and had higher overall physical activity (vector magnitude) and higher M5 and M60 values.

**Table 2.** Novel physical activity metrics in participants from the LIFT (*N* = 337)

Metrics	Values, mean (s.d.)
Vector magnitude (cpm)	2799 (1185)
MVPA (min/day)	17.8 (16.4)
MVPA recommendations (insufficient PA)^a^ (%)	195 (68.4)
M5 (cpm)	40835 (14590)
M60 (cpm)	17023 (7126)
Intensity gradient	−1.80 (0.20)

a Insufficient PA refers to those not meeting the guidelines of 150 min/week MVPA.

### Association of physical activity metrics with fatigue

Results from our multiple linear regression analysis on baseline data are presented in Table 3. In the unadjusted analysis (model 1), step count (*P* < 0.001), vector magnitude (*P* < 0.001), intensity gradient (*P* = 0.002), M5 (*P* = 0.008), M60 (*P* = 0.001), upright time (*P* = 0.012) and MVPA (*P* < 0.001) were negatively associated with CFS. Lying time was positively associated (*P* = 0.005) with CFS. The associations remained, apart from lying time (*P* = 0.056), in model 2. In model 3, vector magnitude remained associated with CFS (*P* = 0.025) but intensity gradient (*P* = 0.111) did not.

**Table 3.** Associations of physical activity metrics with CFS and FSS scores in people with IRDs

Metrics	Model 1		Model 2		Model 3	
	Coefficient (95% CI)	*P*-value	Coefficient (95% CI)	*P*-value	Coefficient (95% CI)	*P*-value
CFS						
Step count	−0.40 (−0.21, −0.59)	**<0.001**	−0.44 (−0.2, −0.69)	**<0.001**		
Vector magnitude	−0.99 (−0.45, −1.53)	**<0.001**	−1.18 (−0.51, −1.85)	**<0.001**	−0.88 (−0.12, −1.64)	**0.025**
Intensity gradient	−5.00 (−1.86, −8.14)	**0.002**	−6.27 (−2.19, −10.35)	**0.003**	−3.75 (0.84, −8.34)	0.111
M5	−0.06 (−0.02, −0.10)	**0.008**	−0.09 (−0.04, −0.14)	**0.0012**		
M60	−0.15 (−0.06, −0.24)	**0.001**	−0.16 (−0.05, −0.27)	**0.003**		
Lying time	1.30 (8.07, −5.47)	**0.005**	8.90 (17.97, −0.17)	0.056		
Sedentary time	0.23 (6.16, −5.70)	0.938	3.82 (12.15, −4.51)	0.370		
Upright time	−7.35 (−1.62, −13.08)	**0.012**	−9.70 (−2.11, −17.29)	**0.013**		
MVPA	−72.09 (−33.14, −111.04)	**<0.001**	−90.75 (−43.00, −138.50)	**<0.001**		
FSS						
Step count	−0.07 (−0.03, −0.11)	**<0.001**	−0.05 (0.00, −0.10)	**0.027**		
Vector magnitude	−0.20 (−0.10, −0.30)	**<0.001**	−0.15 (−0.02, −0.28)	**0.018**	−0.06 (0.08, −0.20)	0.407
Intensity gradient	−1.21 (−0.64, −1.78)	**<0.001**	−1.33 (−0.59, −2.07)	**<0.001**	−1.16 (−2.01, −0.31)	**0.008**
M5	−0.02 (−0.01, −0.03)	**<0.001**	−0.02 (−0.01, −0.03)	**0.003**		
M60	−0.03 (−0.01, −0.05)	**<0.001**	−0.03 (−0.01, −0.05)	**0.008**		
Lying time	1.89 (3.14, 0.64)	**0.003**	1.32 (2.99, −0.35)	0.124		
Sedentary time	−0.19 (0.90, −1.28)	0.738	−0.68 (0.84, −2.20)	0.381		
Upright time	−1.19 (−0.13, −2.25)	**0.029**	−0.35 (1.07, −1.77)	0.625		
MVPA	−16.50 (−9.42, −23.58)	**<0.001**	−14.94 (−6.18, −23.70)	**0.001**		

B-coefficients (95% CI) are presented as × 10^4^ for all variables except intensity gradient. Significant values are in bold.

In the unadjusted analysis (model 1), step count (*P* < 0.001), vector magnitude (*P* < 0.001), intensity gradient (*P* < 0.001), M5 (*P* < 0.001), M60 (*P* < 0.001), upright time (*P* = 0.029) and MVPA (*P* < 0.001) were negatively associated with FSS. Lying time was positively associated (*P* = 0.003) with FFS. The associations remained, apart from lying time (*P* = 0.124) and upright time (*P* = 0.625), in model 2. In model 3, intensity gradient remained associated with FSS (*P* = 0.008) but vector magnitude (*P* = 0.407) did not.

### Effects of PEP on physical activity metrics

Accelerometer data were available for 48 participants in usual care and 45 participants in the PEP at baseline and at the end of the intervention at 6 months; physical activity data at these time points are presented in Table 4. At 6 months there was, relative to usual care, numerically higher values for step count (*P* = 0.071), vector magnitude (*P* = 0.062) and M5 (*P* = 0.087), and upright time was significantly higher in the PEP group (*P* = 0.043).

**Table 4.** Changes in physical activity metrics after 6 months of PEP in people with IRDs

Metrics	UC (*n* = 48)			PEP (*n* = 45)			*P*-value ANCOVA
	Baseline	6 months	Change	Baseline	6 months	Change	
Steps/day	6973 (411)	6763 (3959)	−209 (−678–259)	7455 (2477)	8208 (3831)	772 (−4632008)	0.071
Vector magnitude (cpm)	2815 (1420)	2725 (1452)	−90 (−282–101)	2950 (1026)	3255 (1528)	303 (−168–774)	0.062
Intensity gradient	−1.82 (0.23)	−1.82 (0.24)	0.00 (−0.03–0.05)	−1.74 (0.19)	−1.72 (0.22)	0.02 (−0.04–0.09)	0.275
M5 (cpm)	39 443 (15 875)	39 470 (15 140)	27.0 (−2307–2360)	44 373 (12 579)	46 834 (15 036)	2501 (−884–5886)	0.087
M60 (cpm)	17 247 (9111)	16 653 (9483)	−594 (−1485–297)	17 983 (6746)	19 450 (9593)	1429 (−1598–4456)	0.115
Lying time (min/day)	564.8 (99.6)	577.2 (104.1)	12.4 (−13.3–38.1)	556.5 (75.7)	547.5 (75.8)	−10 (−31–10)	0.103
Sedentary time (min/day)	551.7 (108.3)	549.4 (111.9)	−2.2 (27.4–22.9)	538.8 (109.9)	529.5 (117.1)	−8.1 (−35.5–19.2)	0.556
Upright time (min/day)	323.5 (123.4)	312.9 (120.1)	−10.6 (−25.9–4.7)	344.8 (102.6)	360.8 (103.6)	16.5 (−7.3–40.4)	**0.043**
MVPA (min/day)	18.1 (22.4)	18.3 (23.3)	0.22 (−2.1–2.6)	20.9 (14.5)	24.8 (25.8)	4.4 (−3.2–12.1)	0.237

Data are presented as mean (s.d.). *P*-values are from the comparison to usual care at 6 months after adjustment for baseline values (ANCOVA). Significant values are in bold.

### Association of changes in physical activity metrics with changes in fatigue

After stratifying those in the PEP group into high and low change groups, the change in physical activity metrics and CFS and FSS from baseline to 6 months are presented in Table 5. There was a greater decrease in CFS in the high change group for vector magnitude (*P* = 0.046) and M5 (*P* = 0.009). There was a greater decrease in FSS in the high change group for step count (*P* = 0.003), vector magnitude (*P* < 0.001) and MVPA (*P* = 0.038). No other significant differences between groups were seen.

**Table 5.** Changes in CFS and FSS at 6 months in the PEP group by change in physical activity variables

Metrics	Physical activity variable		CFS			FSS		
	Low change	High change	Low change	High change	*P*-value	Low change	High change	*P*-value
Steps/day	−1770 (2069)	3314 (4004)	−6.7 (6.9)	−8.8 (8.2)	0.122	−0.50 (0.93)	−1.49 (1.59)	**0.003**
Vector magnitude (cpm)	−693 (746)	1300 (1509)	−6.6 (7.2)	−8.9 (8.0)	**0.046**	−0.44 (0.96)	−1.55 (1.53)	**<0.001**
Intensity gradient	−0.14 (0.14)	0.18 (0.14)	−6.5 (6.7)	−8.9 (8.4)	0.150	−0.60 (1.09)	−1.39 (1.55)	0.059
M5 (cpm)	−5934 (6369)	10937 (8017)	−5.2 (6.0)	−10.2 (8.3)	**0.009**	−0.71 (1.30)	−1.28 (1.43)	0.157
M60 (cpm)	−5176 (5529)	8033 (9006)	−7.4 (7.1)	−8.0 (8.2)	0.367	−0.62 (1.23)	−1.37 (1.45)	**0.028**
Lying time (min/day)	−61.1 (34.9)	39.8 (56.6)	−10.1 (8.7)	−5.6 (5.9)	0.062	−1.43 (1.55)	−0.60 (1.10)	0.154
Sedentary time (min/day)	−79.8 (64.0)	63.5 (42.2)	−7.5 (7.1)	−7.9 (8.3)	0.966	−0.92 (1.19)	−1.07 (1.57)	0.675
Upright time (min/day)	−41.3 (47.6)	74.5 (57.4)	−6.1 (7.1)	−9.3 (7.9)	0.150	−0.75 (1.48)	−1.24 (1.26)	0.129
MVPA (min/day)	−8.2 (10.3)	17.0 (29.2)	−6.6 (6.3)	−8.9 (8.7)	0.102	−0.63 (1.18)	−1.36 (1.49)	**0.038**

Data are presented as mean (s.d.). *P*-values from the comparison between low and high change groups by ANCOVA. Significant values are in bold.

## Discussion

Overall, physical activity levels were generally low in our sample of people with IRDs, with almost 70% of people being classified as not meeting the physical activity MVPA recommendations. Our baseline cross-sectional analysis found that several physical activity metrics were associated with fatigue, with some differences comparing CFS and FSS as outcomes. The PEP intervention increased upright time and produced numerically higher measures of total physical activity, relative to usual care, which was associated with greater decreases in both CFS and FSS.

Importantly in the current study, the use of accelerometers, as opposed to self-reported measures, is a strength, as previous work has shown that self-reported methods can underestimate sedentary time and overestimate physical activity [26–28] and that the association of physical activity with health data can be underestimated when self-report measures of physical activity are used [29]. Physical activity levels in the current participants were lower than in the general population [30]. The current physical activity data are similar to previous small studies measuring physical activity by activPAL in people with RA and axial SpA [7, 21] and, as expected, lower than where physical activity was measured by questionnaire [18, 19, 31]. With 68% of our population not meeting the current MVPA recommendations, this highlights that interventions to increase physical activity levels in people with IRDs are needed.

The current study also investigated the distribution of physical activity and its intensity using novel metrics such as the intensity gradient [15]. There is little data to allow a comparison in people with IRDs, but compared with adults from the UK Biobank, the intensity gradient is considerably higher (less negative) in the current study [32]. This may be due to the current study being the first, to our knowledge, to measure the intensity gradient using thigh-worn accelerometers, with previous studies using wrist-worn accelerometers. Alternatively, it could indicate a more even distribution of physical activity across the intensities in our population, i.e. less time spent at higher relative to lower intensities. Further work is clearly needed to investigate this possibility.

In the original LIFT we demonstrated that our PEP intervention, designed to increase physical activity levels, resulted in modest reductions in fatigue severity and impact [14]. These findings are strengthened by our current findings of an association of physical activity levels with fatigue. This is in agreement with previous work that showed, in a broad range of populations, that higher physical activity levels are associated with lower fatigue [6–11]. We extended these findings by showing that overall physical activity is associated with fatigue impact (CFS), whereas the intensity gradient, a measure of the intensity distribution of physical activity, is associated with fatigue severity (FSS). This indicates that tailoring of the physical activity intervention depending on the clinical presentation of fatigue in the patient may enhance its benefits. For example, some populations may benefit simply from more physical activity regardless of intensity, whereas others may experience the most benefit to fatigue symptoms by increasing the time spent in higher intensity activities. However, these data cannot demonstrate causality and so we further explored the follow-up trial data to extend and potentially strengthen these assertions.

In the subgroup of participants with baseline and 6-month accelerometer data, we found a trend for a modest increase in overall physical activity (step count and vector magnitude) with PEPs. Interestingly, in our exploratory analysis, we found associations of improvements in step count, MVPA, M60 and intensity gradient (i.e. more time spent doing activities of higher intensity) (trend), with reductions in FSS and improvements in M5 associated with changes in CFS. These data further support, also in partial agreement with our cross-sectional data, the assertion that different aspects of physical activity are associated with differential changes in fatigue impact and severity. However, it was interesting that changes in the intensity gradient tended to be only associated with changes in FSS, as also shown in our cross-sectional analysis, and so interventions that specifically target a more even distribution of time spent at different physical activity intensities may be of particular benefit for the impact of fatigue. Further work is needed to test this assertion and to investigate the mechanism underlying these observations and how to optimize physical activity interventions to incorporate these findings. In addition, it may be prudent to consider a qualitative evaluation of the PEP to supplement/support these findings based on the questionnaires. Overall, this indicates interventions should probably focus on encouraging overall activity, i.e. moving more regardless of intensity, but it may be worth considering the distribution of activity primarily to target improvements in FSS. While increases in overall physical activity are relatively intuitive, this is not necessarily the case for the intensity gradient. In the current data, a high change in the intensity gradient reflects an increase (less negative) in values. This reflects a more even distribution of time spread across the intensity range, which our data would indicate is better for lowering fatigue and fits with previous concepts around the negatives of boom-and-bust patterns of physical activity (e.g. Hewlett *et al.* [33]).

The current study is not without limitations. This analysis was not pre-specified in our initial statistical analysis plan and should be interpreted with caution as exploratory data. Due to COVID-19 and other technical issues, accelerometer data collection was partial, so this analysis is based on a small number of participants, resulting in a level of uncertainty in our conclusions. On top of this, it is likely that the changes in physical activity (as we have measured) induced by PEPs do not fully explain the observed improvements in fatigue, suggesting that PEPs may also have had other benefits. It is possible that PEPs also, independent of changes in physical activity, improved sleep, cognition and mood, which may influence fatigue.

In conclusion, the current data highlight the importance of physical activity for management of fatigue in people with IRDs and that different aspects of physical activity may have differentially influenced fatigue severity and impact. With physical activity levels generally low, the current study shows that the PEP is an important strategy for managing fatigue in people with IRDs. However, further work is needed to develop and optimize the components of the PEP to target the symptoms and impact of fatigue.

## Supplementary Material

rkae106_Supplementary_Data

## Data Availability

Anonymized individual patient data will be made available following any reasonable request made to the corresponding author, subject to a data sharing agreement and UK research governance regulations. The intervention manuals can be found at https://www.abdn.ac.uk/iahs/research/epidemiology/lift-1286.php.
