# Supplementary material for: Associations of physical activity levels with fatigue in people with inflammatory rheumatic diseases in the LIFT trial
Source: Rheumatol Adv Pract. 2024 Aug 24;8(3):rkae106. doi: 10.1093/rap/rkae106 (PMC11384110; doi:10.1093/rap/rkae106)
Supplement: rkae106_Supplementary_Data [file rkae106_supplementary_data.docx]

**
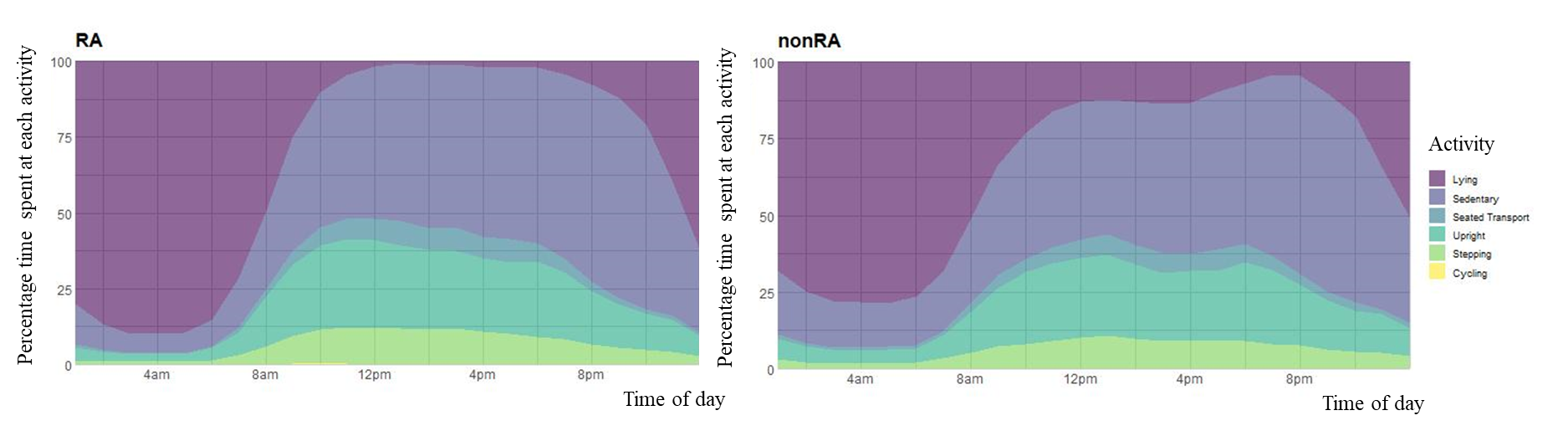
Supplementary Figure S1 Variation in accelerometer measured time by activity type in participants in the LIFT stratified into patients with and without rheumatoid arthritis (RA)**

**
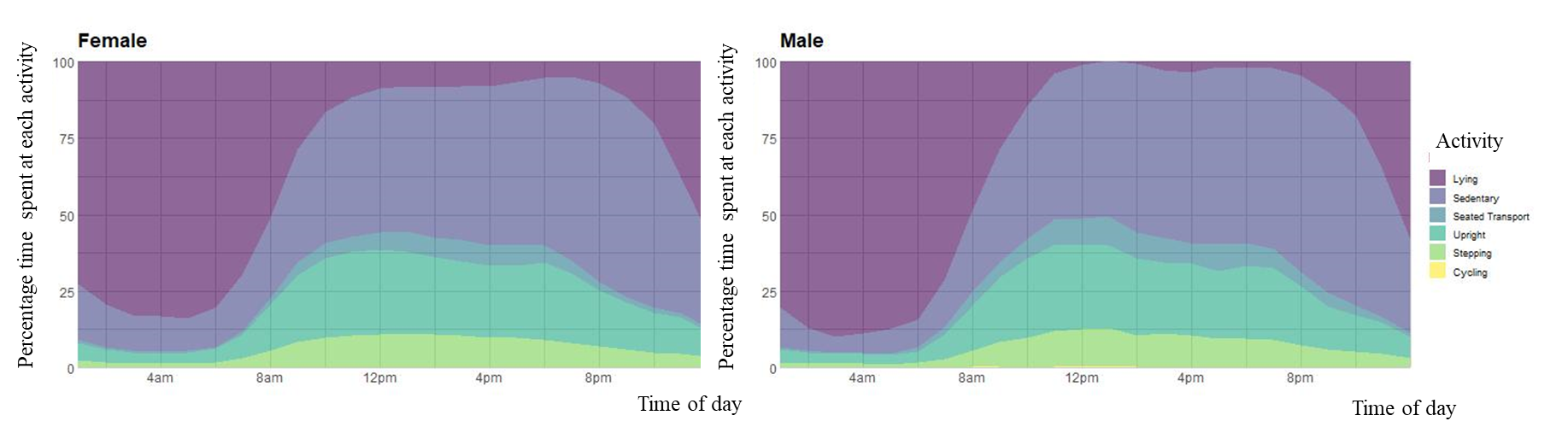
Supplementary Figure S2 Variation in accelerometer measured time by activity type in participants in the LIFT stratified by sex**

**
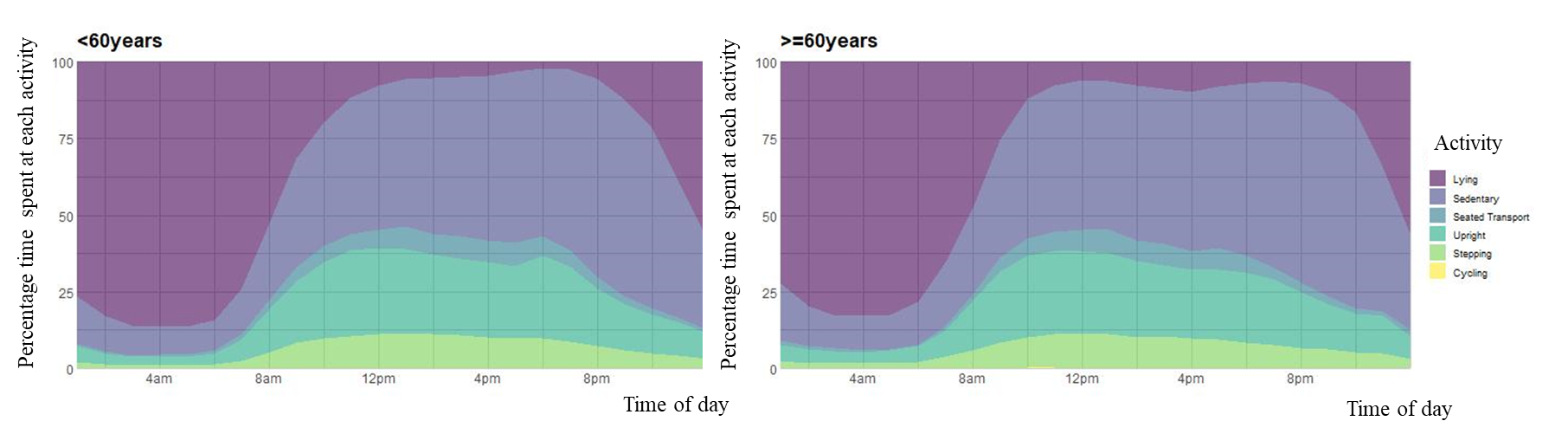
Supplementary Figure S3 Variation in accelerometer measured time by activity type in participants in the LIFT trial stratified by age**

**Supplementary Table S1 Basic demographic and physical activity variables in participants from the LIFT trial stratified into patients with and without Rheumatoid Arthritis (RA)**

|  | **RA (n=191)** | **Non-RA (n=146)** | **P value** |
| --- | --- | --- | --- |
| **Age (years)** | 59.7 (12.0) | 55.5 (13.0) | **0.003** |
| **Body Mass (kg)** | 78.2 (16.8) | 78.7 (16.0) | 0.812 |
| **Diagnosis (N^o^ non-RA (%))** | 42 (22.0) | 40 (27.4) | 0.309 |
| **Step Count (steps/day)** | 6990 (3339) | 6919 (3165) | 0.855 |
| **Activity Score (MET.s^-1^)** | 83.3 (3.6) | 83.3 (3.4) | 0.952 |
| **Sedentary Time (min/day)** | 551.6 (111.1) | 546.3 (110.6) | 0.689 |
| **Upright Time (min/day)** | 321.7 (111.0) | 331.2 (114.9) | 0.484 |
| **Stepping Time (min/day)** | 91.5 (38.0) | 91.0 (39.0) | 0.921 |
| **Cycling Time (min/day)** | 0.91 (3.17) | 0.72 (2.40) | 0.576 |
| **Lying Time (min/day)** | 564.7 (80.1) | 567.2 (85.2) | 0.796 |
| **Seated Transport (min/day)** | 49.1 (34.9) | 54.2 (45.6) | 0.279 |

*Data are mean (SD). Bold text indicates significant P values.*

**Supplementary Table S2 Basic demographic and physical activity variables in participants from the LIFT trial stratified by sex**

|  | **Female (n=255)** | **Male (n=82)** | **P value** |
| --- | --- | --- | --- |
| **Age (years)** | 57.6 (12.9) | 58.8 (11.6) | 0.456 |
| **Body Mass (kg)** | 75.2 (16.3) | 87.1 (13.1) | **<0.001** |
| **Diagnosis (N^o^ non-RA (%))** | 106 (41.6) | 40 (48.8) | 0.309 |
| **Step Count (steps/day)** | 6916.7 (3318.4) | 7090.6 (3089.4) | 0.699 |
| **Activity Score (MET.s^-1^)** | 83.3 (3.6) | 83.4 (3.3) | 0.826 |
| **Sedentary Time (min/day)** | 547.2 (119.6) | 555.8 (77.8) | 0.574 |
| **Upright Time (min/day)** | 328.7 (115.6) | 317.0 (103.2) | 0.451 |
| **Stepping Time (min/day)** | 90.3 (38.1) | 94.3 (38.8) | 0.447 |
| **Cycling Time (min/day)** | 0.54 (2.20) | 1.70 (4.2) | **0.003** |
| **Lying Time (min/day)** | 567.6 (81.3) | 560.3 (85.4) | 0.525 |
| **Seated Transport (min/day)** | 45.9 (31.3) | 67.9 (56.0) | **<0.001** |

*Data are mean (SD). Bold text indicates significant P values.*

**Supplementary Table S3 Basic demographic and physical activity variables in participants from the LIFT trial stratified by age**

|  | **< 60 years (n=178)** | ≥ **60 years (n=159)** | **P value** |
| --- | --- | --- | --- |
| **Age (years)** | 48.1 (8.3) | 68.7 (5.8) |  |
| **Body Mass (kg)** | 79.0 (17.3) | 77.4 (14.8) | 0.492 |
| **Sex (number male (%))** | 41 (23.0) | 41 (25.8) | 0.645 |
| **Step Count (steps/day)** | 6725 (3342) | 6959 (3259) | 0.255 |
| **Activity Score (MET.s^-1^)** | 83.0 (3.6) | 83.3 (3.5) | 0.209 |
| **Sedentary Time (min/day)** | 577.5 (116.9) | 549.3 (110.7) | **<0.001** |
| **Upright Time (min/day)** | 312.6 (112.8) | 325.8 (112.6) | 0.063 |
| **Stepping Time (min/day)** | 89.4 (39.9) | 91.3 (38.3) | 0.435 |
| **Cycling Time (min/day)** | 0.88 (3.10) | 0.83 (2.86) | 0.784 |
| **Lying Time (min/day)** | 554.5 (80.3) | 565.8 (82.2) | 0.363 |
| **Seated Transport (min/day)** | 58.4 (42.7) | 43.3 (34.9) | **0.001** |

*Data are mean (SD). Bold text indicates significant P values.*

**Supplementary Table S4 Novel physical activity metrics in participants from the LIFT trial stratified into patients with and without Rheumatoid Arthritis (RA)**

|  | **RA (n=191)** | **Non-RA (n=146)** | **P value** |
| --- | --- | --- | --- |
| **Vector Magnitude (cpm)** | 2781 (1199) | 2823 (1170) | 0.770 |
| **MVPA (min/day)** | 17.9 (17.6) | 17.6 (14.7) | 0.869 |
| **MVPA recommendations (number inactive (%))** | 110 (67.9) | 85 (69.1) | 0.930 |
| **M5 (cpm)** | 40294 (14738) | 41548 (14421) | 0.473 |
| **M60 (cpm)** | 17130 (7408) | 16883 (6763) | 0.773 |
| **IG Slope** | -1.81 (0.21) | -1.79 (0.20) | 0.485 |

*Data are mean (SD).*

**Supplementary Table S5 Novel physical activity metrics in participants from the LIFT trial stratified by sex**

|  | **Female (n=255)** | **Male (n=82)** | **P value** |
| --- | --- | --- | --- |
| **Vector Magnitude (cpm)** | 2697 (1160) | 3113 (1213) | **0.011** |
| **MVPA (min/day)** | 18.1 (17.35) | 16.9 (12.9) | 0.608 |
| **MVPA recommendations (number inactive (%))** | 149 (69.3) | 46 (65.7) | 0.680 |
| **M5 (cpm)** | 40161 (15320) | 42904 (11936) | 0.172 |
| **M60 (cpm)** | 16552 (7332) | 18471 (6279) | **0.050** |
| **IG Slope** | -1.81 (0.22) | -1.76 (0.16) | 0.080 |

*Data are mean (SD). Bold text indicates significant P values.*

**Supplementary Table S6 Novel physical activity metrics in participants from the LIFT trial stratified by age**

|  | **< 60 years (n=178)** | ≥ **60 years (n=159)** | **P value** |
| --- | --- | --- | --- |
| **Vector Magnitude (cpm)** | 2962 (1189) | 2615 (1156) | **0.001** |
| **MVPA (min/day)** | 19.4 (17.5) | 16.0 (14.9) | 0.083 |
| **MVPA recommendations (number inactive (%))** | 96 (71.6) | 99 (65.6) | 0.330 |
| **M5 (cpm)** | 43548 (13292) | 37779 (15412) | **0.001** |
| **M60 (cpm)** | 18018 (7640) | 15902 (6341) | **0.012** |
| **IG Slope** | -1.79 (0.20) | -1.81 (0.21) | 0.342 |

*Data are mean (SD). Bold text indicates significant P values.*
